# Detecting human melanoma cell re-differentiation following BRAF or heat shock protein 90 inhibition using photoacoustic and magnetic resonance imaging

**DOI:** 10.1038/s41598-017-07864-8

**Published:** 2017-08-15

**Authors:** Anant Shah, Teresa Delgado-Goni, Teresa Casals Galobart, Slawomir Wantuch, Yann Jamin, Martin O. Leach, Simon P. Robinson, Jeffrey Bamber, Mounia Beloueche-Babari

**Affiliations:** 1 Cancer Research UK Cancer Imaging Centre, The Institute of Cancer Research, London and The Royal Marsden NHS Foundation Trust, Sutton, London SM2 5PT United Kingdom; 2 Joint Department of Physics, Division of Radiotherapy and Imaging, The Institute of Cancer Research, London and The Royal Marsden NHS Foundation Trust, Sutton, London SM2 5PT United Kingdom

## Abstract

Targeted therapies specific to the BRAF-MEK-ERK signaling pathway have shown great promise in the treatment of malignant melanoma in the last few years, with these drugs now commonly used in clinic. Melanoma cells treated using these agents are known to exhibit increased levels of melanin pigment and tyrosinase activity. In this study we assessed the potential of non-invasive imaging approaches (photoacoustic imaging (PAI) and magnetic resonance imaging (MRI)) to detect melanin induction in SKMEL28 human melanoma cells, following inhibition of Hsp90 and BRAF signaling using 17-AAG and vemurafenib, respectively. We confirmed, using western blot and spectrophotometry, that Hsp90 or BRAF inhibitor-induced melanoma cell differentiation resulted in an upregulation of tyrosinase and melanin expression levels, in comparison to control cells. This post-treatment increase in cellular pigmentation induced a significant increase in PAI signals that are spectrally identifiable and shortening of the MRI relaxation times *T*_1_ and ${{\boldsymbol{T}}}_{{\bf{2}}}^{{\boldsymbol{\ast }}}$. This proof-of-concept study demonstrates the potential of MRI and PAI for detecting the downstream cellular changes induced by Hsp90 and BRAF-MEK-targeted therapies in melanoma cells with potential significance for *in vivo* imaging.

## Introduction

Malignant melanoma is an aggressive form of skin cancer that has shown an increased rate of incidence especially amongst the Caucasian population over the past few years^1^. The discovery of the prevalence of BRAF mutation in this disease (≈50% of cases) and the role of the oncoprotein, particularly the highly active V600E variant, in the onset and progression of melanoma^2, 3^ have fuelled much interest in targeting the BRAF-MEK-ERK pathway for melanoma treatment. Indeed, several inhibitors of BRAF and MEK are now approved for the treatment of BRAF-driven melanoma (e.g. the BRAF inhibitors vemurafenib, dabrafenib and the MEK inhibitor trametinib)^4–6^ and many more are in development for use either as single agents or in combination with other anti-cancer drugs^7^.

Another approach for the downregulation of BRAF activity is direct degradation of the protein through inhibition of heat shock protein 90 (Hsp90). Hsp90 is a molecular chaperone involved in maintaining the conformational stability of many oncogenic clients including BRAF, CRAF, AKT and HER2^8–10^. Inhibition of Hsp90 with agents such as 17-allylamino-17-demethoxygeldanamycin (17-AAG) and AUY922 results in proteasomal degradation of the client proteins and consequently simultaneous blockade of multiple key oncogenic signal transduction pathways, including the BRAF-MEK-ERK1/2 and PI3K-AKT, which then leads to inhibition of proliferation, invasion, angiogenesis and induction of tumour cell redifferentiation^8–13^. Hsp90 inhibitors have shown activity in several pre-clinical models of melanoma and are currently undergoing clinical testing in several tumour types including malignant melanoma^14–16^.

Detecting pharmacodynamic (PD) biomarkers of the action of BRAF-MEK-ERK and Hsp90 inhibitors is key for monitoring target blockade and modulation of downstream cellular processes in addition to probing therapeutic efficacy^17–19^. In this regard, non-invasive imaging biomarkers of the downstream consequences of target modulation are highly desirable as they remove the need for surgical intervention and afford the possibility of performing longitudinal studies in the same patient^20^. In particular, molecular and functional imaging biomarkers that can inform on therapeutic activity prior to any measurable changes in tumour size are extremely valuable. Furthermore, a non-invasive early indication of whether the treatment has “hit the target” (i.e., in addition to measures of whether the tumour is responding) would allow more effective patient management than is currently possible.

In this study we used photoacoustic imaging (PAI) and magnetic resonance imaging (MRI) to assess the PD biomarkers of the downstream cellular changes that follow the inhibition of Hsp90 and BRAF signaling in melanoma cells. PAI enables the display of optical absorption contrast with a high (sub-mm) resolution at depths of up to a few centimetres^21^. Multiwavelength PAI can provide information on tissue composition by identifying spectral signatures of a wide range of endogenous chromophores, such as melanin^22^, lipids^23, 24^, collagen^25^, myoglobin^26^, oxygenated and deoxygenated haemoglobin^27^. MRI allows the non-invasive probing of tumour physiology providing structural as well as functional information including cellular density and tissue relaxation parameters such as *T*_1_, *T*_2_ and ${T}_{2}^{\ast }$, which are sensitive to changes in paramagnetic molecular species content^28^. These measurements are relevant in understanding water molecular dynamics in biologic systems, as they depend on tissue microstructure and the chemical and physical environments of water protons in it^29^. Paramagnetic species (free metals and radicals), such as those scavenged by melanin alter tissue local magnetic environment, producing local magnetic fields, shortening the original relaxation parameters^30^. Previous work has shown that forced expression of tyrosinase in human breast cancer cells *in vitro* results in increased melanin content, increased photoacoustic (PA) signals at 532 nm^31^ and 650 nm^32^, and a reduction in *T*_1_^31, 32^ and *T*_2_^31^ MRI relaxation times upon exogenous administration of iron.

**Figure 1:**
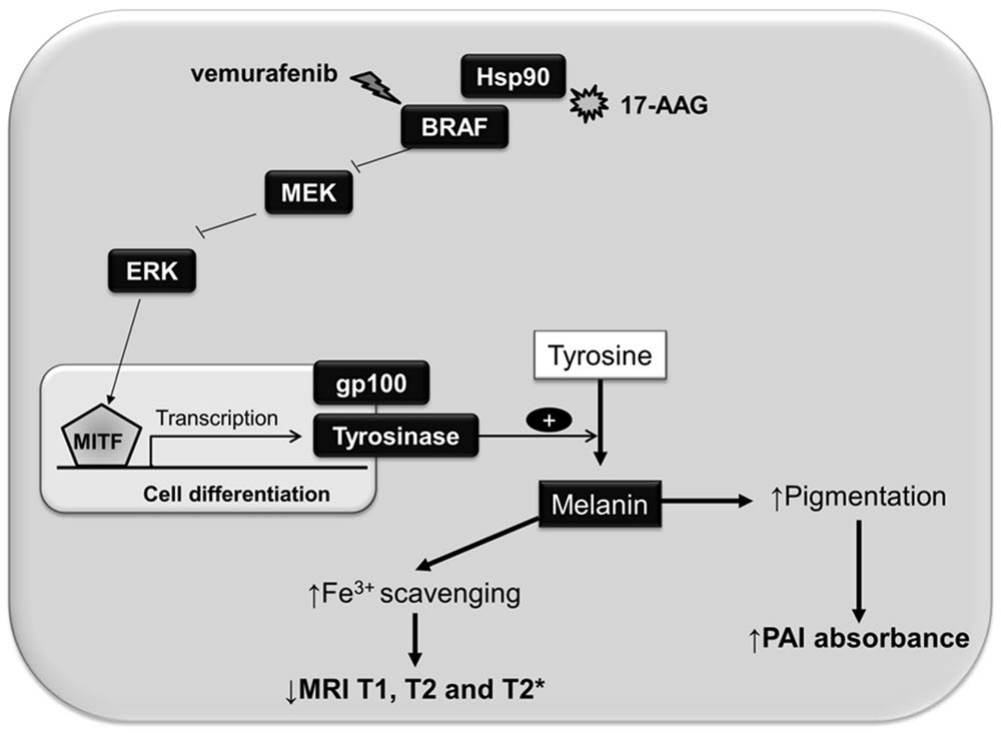
Schematic representation of the molecular basis for the induction in melanin and generation of MRI and PAI-based contrast following exposure of BRAF mutant human melanoma cells to BRAF and Hsp90 inhibitors.

In this study, we posited that Hsp90 or BRAF inhibitor-induced melanoma cell differentiation will result in increased cellular melanin levels through upregulation of tyrosinase expression and that this in turn should lead to a) increased optical absorption detectable by PAI and b) a reduction in *T*_1_, *T*_2_ and ${T}_{2}^{\ast }$ relaxation times detectable by MRI, as depicted in Fig. 1. This hypothesis was tested in BRAF mutant SKMEL28 human melanoma cells following treatment with the Hsp90 inhibitor 17-AAG and the BRAF inhibitor vemurafenib.

## Results

### Hsp90 and BRAF inhibitor-induced human melanoma cell differentiation leads to increased pigmentation

Prior to conducting the cell imaging experiments, we first assessed the drug-induced molecular and cellular effects to confirm the expected phenotypic changes. Treatment of SKMEL28 human melanoma cells with the Hsp90 inhibitor 17-AAG (50 nM) or the BRAF inhibitor vemurafenib (1 *μ*M) for 72 h led to a reduction in cell counts to 37% ± 6% and 47% ± 4% of controls, respectively (p < 0.001).

**Figure 2:**
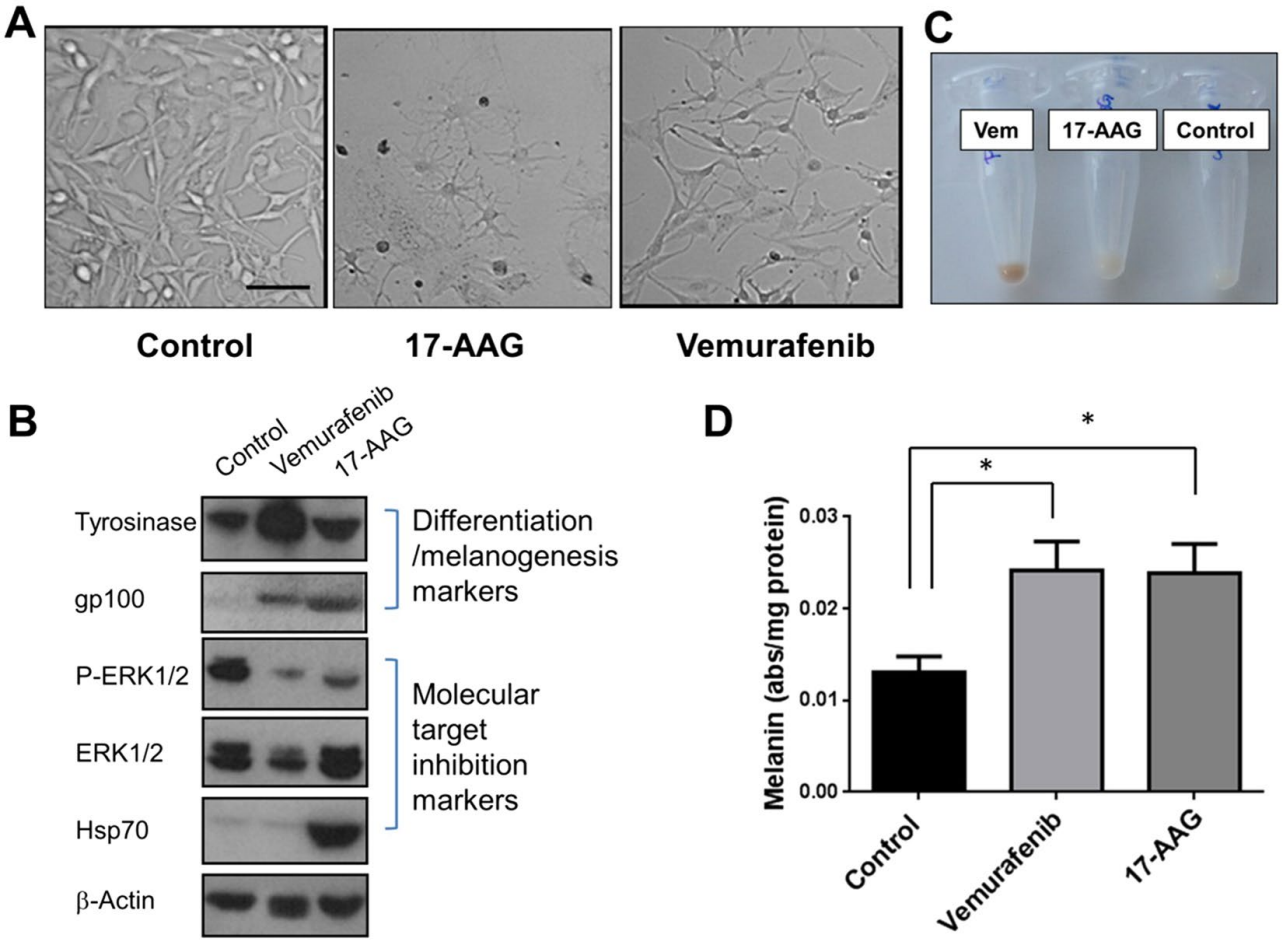
Hsp90 and BRAF inhibition leads to human melanoma cell differentiation and induction of melanin synthesis. (**A**) Bright field images showing the increased dendritic morphology in SKMEL28 melanoma cells following inhibition with vemurafenib (1 *μ*M) or 17-AAG (50 nM) for 72 h (scale bar: 100 *μ*m). (**B**) Western blots confirm the induction of melanoma cell differentiation (increased gp100) and melanogenesis (increased tyrosinase) concomitant with the expected molecular signature of BRAF and Hsp90 inhibition following exposure to vemurafenib (Vem) and 17-AAG for 72 h. (**C**) Images of SKMEL28 pellets showing increased pigmentation following exposure to vemurafenib or 17-AAG for 72 h. The different tubes contain the same number of cells. (**D**) Spectrophotometric absorbance measurements in SKMEL28 cells confirm induction of melanin synthesis following treatment with the BRAF inhibitor vemurafenib and the Hsp90 inhibitor 17-AAG.

As shown in Fig. 2A, light microscopy revealed stark changes in cell morphology following exposure to the Hsp90 and BRAF inhibitors, with the adoption of a dendritic appearance characteristic of melanoma cell re-differentiation^33^. Western blot analysis of cellular proteins showed reduced ERK phosphorylation following vemurafenib and 17-AAG treatment together with an increase in Hsp70 in 17-AAG-treated cells, consistent with the expected molecular effects of Hsp90 and BRAF-MEK-ERK signaling inhibition^34, 35^. These drug-induced effects were paralleled with induction of tyrosine and gp100 protein expression under both treatments, confirming induction of melanoma cell re-differentiation (Fig. 2B). Inspection of the colour of cell pellets (containing the same number of cells each) indicated that drug treatment with either 17-AAG or vemurafenib led to increased cell pigmentation (browner colour) consistent with increased melanin content (Fig. 2C). Further, measurement of intracellular melanin content revealed increased levels in both 17-AAG and vemurafenib-treated SKMEL28 cells up to 2-fold relative to controls (Fig. 2D). Thus, at the concentration and exposure time used in our experiments, treatment with the Hsp90 inhibitor 17-AAG and the BRAF inhibitor vemurafenib led to induction of melanoma cell re-differentiation and increased cellular melanin levels.

### Human melanoma cell re-differentiation is associated with increased PAI signal

**Figure 3:**
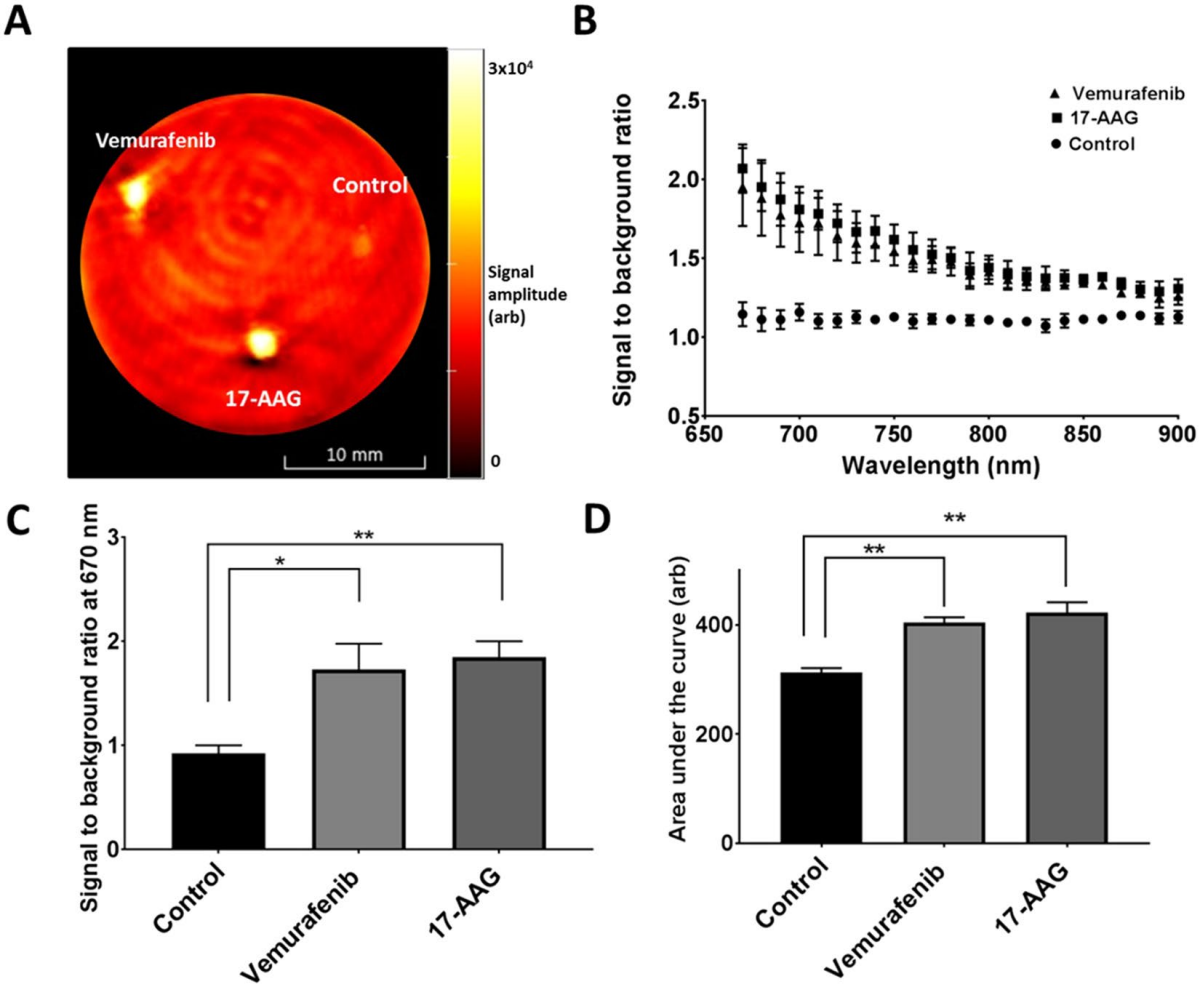
Changes in PAI signals following melanoma cell differentiation. (**A**) Optoacoustic image of a phantom with the control and treated cell inclusions at 670 nm absorption. Circular inclusions containing control cells, and cells treated with 17-AAG and vemurafenib to the right, bottom and left of the phantom, respectively (scalebar: 10 mm). (**B**) Signal to background ratio plot of the untreated cells (●) and cells treated with 17-AAG ($\blacksquare$) and vemurafenib ($\blacktriangle$), obtained by measuring the ratio of mean PA signal intensities of the inclusions to the background, at a wavelength interval of 10 nm from 670 nm–900 nm. The error bars represent standard error of mean (n = 3). (**C**) Bar chart comparing the signal to background ratio at 670 nm, between control and treated cells. (**D**) Comparison of area under the curve of the SBR plots between the treated and control cells. The asterisk sign indicates the significant difference between the mean of the groups (*p < 0.05, **p < 0.01).

Next, we used PAI to assess the optical absorbance properties of control and re-differentiated SKMEL28 human melanoma cells. The photoacoustic signals of treated and control SKMEL28 cell pellet inclusions, embedded in an agar phantom, were measured for wavelengths ranging from 670 nm–900 nm. Figure 3A depicts a photoacoustic image of a representative phantom imaged at 670 nm, showing inclusions containing control cells, and cells treated with 17-AAG and vemurafenib. Since, the treated cells express greater levels of melanin compared to controls, consequent generation of PA contrast was expected. Accordingly, an extremely weak signal was observed from the inclusion of control cells, in contrast to the treated cell inclusions, which were visible in both 17-AAG- and vemurafenib-exposed cells.

Figure 3B shows a plot of measured PA signal to background ratio (SBR) with respect to wavelength (i.e. a relative absorbance spectrum) for each inclusion. The SBR spectrum for the treated cell inclusions corresponds to the established absorption signature of melanin^36^, confirming an increased level of cellular melanin in response to treatment. Treatment with vemurafenib and 17-AAG caused an approximately two-fold increase in the SBR at 670 nm (Fig. 3C, p = 0.036 and p = 0.0057, respectively) and an approximately 1.3 fold increase in the area under the curve of SBR spectra (Fig. 3D, p = 0.0017 and p = 0.0063, respectively), in comparison to the untreated cells. The degree of increase in treated cell optical absorbance was similar for both drugs, consistent with the similar levels of increase in intracellular melanin induced post-treatment.

### Induction of human melanoma cell pigmentation is associated with shortening of MRI relaxation times T1 and T2*

**Figure 4:**
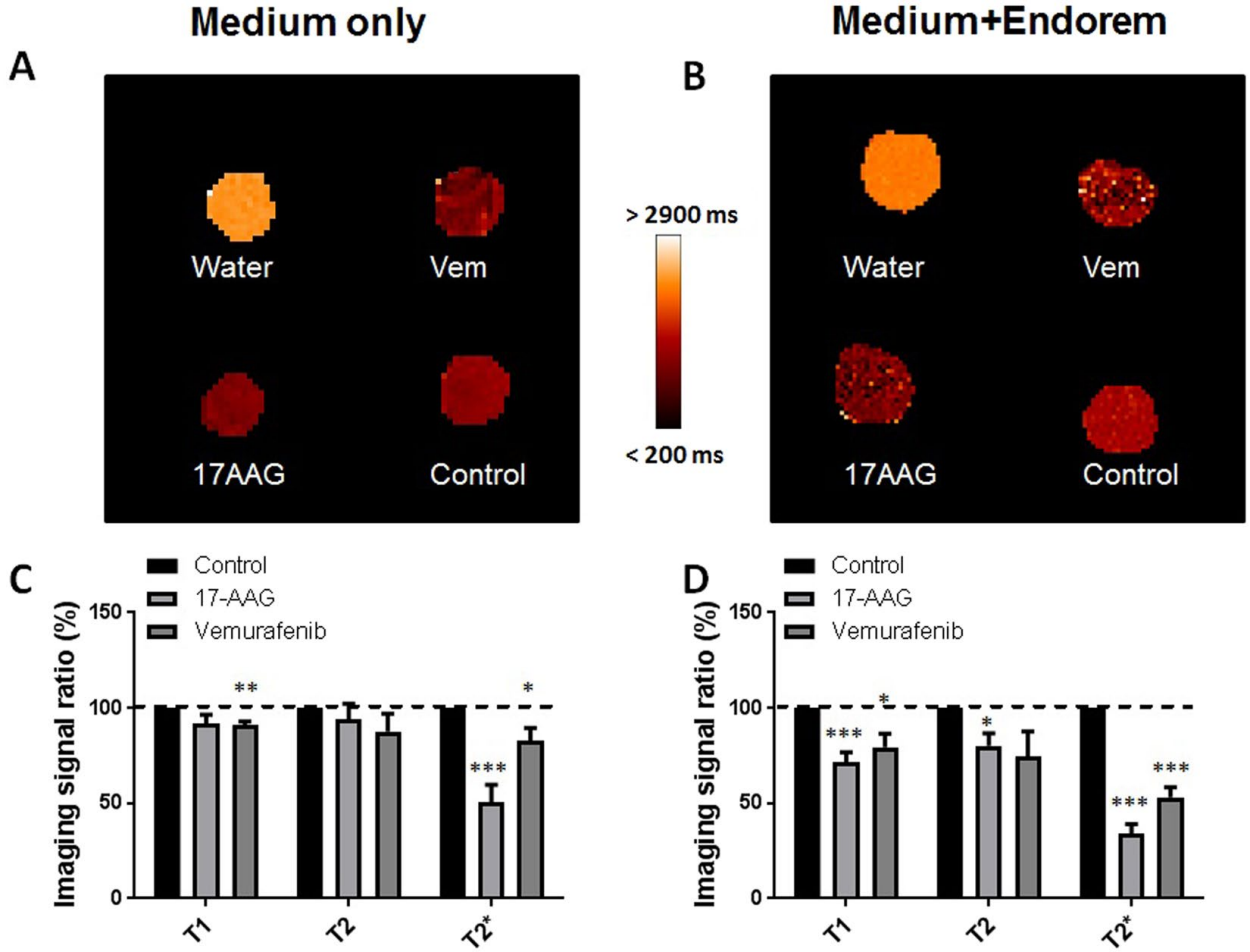
MRI relaxation time changes associated with melanin accumulation under BRAF and Hsp90 inhibition. (**A**,**B**) Show *T*_1_ maps obtained from 4 eppendorf tubes containing water as an external control and 3 different SKMEL28 cell pellets: DMSO treated cells (Cont), cells under Hsp90 inhibition (17-AAG) and cells under BRAF inhibition (Vem). Cells were cultured in standard medium (**A**) or medium containing exogenous iron oxide (Endorem, 0.5 *μ*g/ml) (**B**), which enhanced differences in *T*_1_ between treated and control pellets as shown in the maps. (**C**,**D**) Show the changes in *T*_1_, *T*_2_ and ${T}_{2}^{\ast }$ relaxation times of treated cells with respect to control, cultured in standard medium (**C**) and medium containing Endorem (**D**). Both drug treatments induce a significant shortening in *T*_1_ and ${T}_{2}^{\ast }$ values, which is enhanced in cells cultured with Endorem. The error bars represent standard error of the mean (n = 5). (*p < 0.05, **p < 0.01, ***p < 0.001).

Melanin is a scavenger of iron, thus increased cell pigmentation is expected to lead to increased iron chelation leading to a shortening of the MRI relaxation times *T*_1_, *T*_2_ and ${T}_{2}^{\ast }$. To assess the ability of MRI to detect such effects, we monitored MRI image contrast obtained from cells grown in standard media as well as media supplemented with exogenous superparamagnetic iron oxide particles. Representative *T*_1_ maps are displayed in Fig. 4A, showing differences in contrast between control and treated cells, which was more pronounced in cells exposed to exogenous iron oxide particles.

Imaging data processing indicated that drug treatment of cells grown in standard media had no significant effect on longitudinal relaxation time *T*_1_ but induced lower transverse relaxation time ${T}_{2}^{\ast }$ values, relative to controls (which also incorporates local susceptibility effects, in this case iron binding-induced field inhomogeneity), in 17-AAG-treated cells. In contrast, vemurafenib-treated cells showed a significantly lower *T*_1_ and ${T}_{2}^{\ast }$, relative to controls (Fig. 4A,C). Supplementation of growth media with exogenous iron oxide enhanced the image contrast between control and treated cells and led to a significant shortening of the MRI relaxation times *T*_1_ and ${T}_{2}^{\ast }$ in both 17-AAG- and vemurafenib-treated cells (Fig. 4B,D). Thus, increased cell pigmentation following BRAF or Hsp90 inhibitor treatment leads to increased iron chelation, resulting in shortening of *T*_1_ and ${T}_{2}^{\ast }$ MRI relaxation times that is enhanced with exogenous iron supplementation.

As a preliminary indication of the relative sensitivity of PAI and MRI to the changes induced in the melanin content of the cells, we estimated the coeffiecient of variation (CV) values of the ratio of imaging signal in the treated cells to that in the controls. The CV values for PAI were 0.186 for treatment with vemurafenib and 0.141 for treatment with 17-AAG, whereas the same values for MRI ${T}_{2}^{\ast }$ (the best performing relaxation time) were 0.186 and 0.419, respectively.

## Discussion

Molecularly targeted approaches to the treatment of malignant melanoma have in recent years seen unprecedented advances, with drugs targeting BRAF-MEK-ERK signaling now in routine clinical use, including the BRAF inhibitor vemurafenib^37^ and many more undergoing clinical development.

Developing biomarkers of drug activity is crucial for probing target modulation and treatment efficacy and hence clinical follow up^17, 18^, with minimally invasive biomarkers of treatment response providing obvious advantages^20^. In this study, we set out to investigate the feasibility of using two imaging approaches in monitoring the downstream cellular consequences of treatment with BRAF and Hsp90 inhibitors in BRAF mutant human melanoma cells. Specifically, we explored the role of non-invasive imaging with PAI and MRI in detecting melanoma cell functional differentiation following treatment with the prototype Hsp90 inhibitor 17-AAG and the clinically relevant BRAF inhibitor vemurafenib. These agents cause decreased MEK-ERK1/2 signaling, inhibition of cell proliferation and induction of human melanoma cell differentiation characterised by increased cell pigmentation through induction of melanin synthesis and deposition enzymes such as tyrosinase^38, 39^. Thus we hypothesised that PAI and MRI may be amenable to the detection of such effects through melanin-generated imaging contrast.

Our findings show that inhibition of Hsp90 and BRAF signaling in human melanoma cells is, as expected, associated with induction of cell differentiation that leads to increased cell pigmentation characterised by increased tyrosinase protein expression and melanin content. Importantly, we show that these changes are detectable by non-invasive cell imaging data, translating to significantly increased PAI signals and significant shortening of the MRI relaxation time ${T}_{2}^{\ast }$, relative to controls. Calculations of CV show that PAI and MRI were of roughly equal sensitivity in detecting melanin expression due to treatment with vemurafenib but that PAI may have been more sensitive than MRI in detecting changed expression due to treatment with 17-AAG. This suggests that PAI might have equivalent or better sensitivity compared to MRI at 7 T, and might be more advantageous compared to MRI at clinical field strengths. However, further work is needed to carry out a thorough sensitivity comparison of the two methods.

Whilst our data demonstrate the potential of PAI and MRI in detecting melanoma cell re-differentiation post Hsp90 and BRAF inhibition, each modality has its own merits and demerits for clinical use. MRI is a well-established imaging modality, routinely used in clinical practice. It is capable of producing detailed images of the anatomical structure of all parts of the body, but the financial cost of these images is high. PAI, on the other hand, is a relatively new imaging modality that holds great promise of clinical translation. The main limitation of PAI is its limited imaging depth of up to 5–6 cm^40^. However, PAI offers examination convenience as well as reduced cost, and the ability to easily integrate the result in a multivariate approach with other ultrasound modalities. Several clinical PAI systems existing in a research context have demonstrated the potential of PAI in imaging cutaneous^41, 42^ and ocular melanoma^43^. Stoffels *et al*.^44^ demonstrated the clinical potential of PAI in determining the metastatic status of sentinel lymph nodes in melanoma, located up to a depth of 5 cm, utilising a commercially available MSOT system. Our results suggest that PAI could potentially visualise the downstream cellular changes, post treatment, in such locations (cutaneous, mucosal and ocular melanoma, and metastatic melanoma in lymph nodes and subcutaneous tissues). Given that both the imaging modalities have their own advantages and limitations, and can provide a range of complementary information, some similar (for example haemoglobin content and blood oxygenation) and some unique (for example, MRI apparent diffusion coefficient values related to tissue cell density), a carefully planned imaging approach dependent on the nature, location and spread of the disease, should permit the selection of either one or both modalities to image the changes in melanoma cell re-differentiation following BRAF or Hsp90 inhibition.

However, implementation of this approach for *in vivo* imaging would be accompanied with additional challenges. The presence of other tissue components, primarily haemoglobin, could introduce complexities in detecting the increased expression of melanin using PAI and MRI. The paramagnetic nature and absorption of light by oxy- and deoxy-haemoglobin would necessitate the implementation of approaches to enable differentiation of the MRI and PAI signals of melanin from oxy- and deoxy-haemoglobin. For PAI, this complexity could be addressed by using spectral unmixing algorithms that could differentiate and detect the unique optical absorption signatures of melanin, oxy- and deoxy-haemoglobin^45, 46^. An approach for accounting for the depth and wavelength-dependence of attenuation of light in tissue^47^, and heterogenous acoustic properties of tissues^48^, might be essential for accurate quantification of an increase in melanin expression, post-treatment. For MRI, deoxygenated blood generates a very similar contrast to melanin. However, the distribution of oxy- and deoxy- haemoglobin could be imaged and distinguished from melanin using intrinsic susceptibility MRI with hyperoxia^49^ as unlike haemoglobin, melanin would not be sensitive to changes in blood oxygenation. The changes observed by MRI were enhanced with the addition of exogenous iron, consistent with the mechanism underlying the image contrast. However, under baseline conditions, changes in image contrast could also be observed between control and treated cells indicating the potential to detect these differences in tumours based on endogenous contrast. This is also the case for the imaging contrast differences observed with PAI, further emphasising the value of the approach used here.

Upregulation of melanoma differentiation markers is observed in patients’ tumours following treatment with vemurafenib, and was associated with disease evolution, being downregulated upon progression. Thus, our findings could have significance not only for monitoring drug-induced pathway modulation but also for assessing tumour responsiveness to therapy. More importantly, and of particular relevance to current developments in melanoma, drug-induced upregulation of melanocyte differentiation antigens facilitates immune recognition of melanoma cells and correlates with increased tumour infiltration by *CD*8+ cytotoxic T lymphocytes^50^. Similar effects have also been described with Hsp90 inhibitors in pre-clinical models^51^. Thus, an imaging assay such as the one proposed here should enable stratification of patients who are more likely to benefit from combination treatment with BRAF-MEK-ERK inhibitors plus immunotherapy, currently under evaluation in several clinical trials^52^.

Further work will be required to assess the potential of PAI and MRI in detecting melanoma tumour differentiation in additional melanoma tumour models with different levels of baseline pigmentation, and assessing the feasibility of our approach *in vivo*. Nevertheless, this study provides proof-of-concept for the further study of these biomarkers as non-invasive imaging tools to report on the downstream cellular consequences of Hsp90 and BRAF-MEK-targeted therapies in melanoma models *in vivo* with potential significance for patient follow up and management.

## Methods

### Cell culture

Human malignant melanoma SKMEL28 cells (harbouring mutant *BRAF* ^*V*600*E*^) were originally purchased from the American Type Culture Collection and authenticated with STR profiling in-house on 16th October 2015. Cells were cultivated in DMEM containing 10% (v/v) heat inactivated fetal calf serum, 100 U/ml penicillin and 100 g/ml streptomycin (Life Technologies; Paisley, UK), passaged for no longer than 3 months at a time and regularly screened for mycoplasma.

### Cell preparation for imaging analyses

Logarithmically growing SKMEL28 were treated with 50 nM 17-AAG or 1 *μ*M vemurafenib for 72 h to achieve induction of cell differentiation and a ca. 50% reduction in cell counts. Control cells in all experiments were exposed to DMSO at a concentration of 0.01%. For PAI, cells were embedded in a cylindrical, tissue mimicking agarose gel phantom of approximately 25 mm diameter and 60 mm height. An open ended syringe (50 ml in volume, 26 mm inner diameter) was filled up with 15 ml of agar solution, composed of agar (Sigma, 1.5% by weight) and 0.5% Intralipid® for ultrasonic scattering. The solution was allowed to solidify at room temperature and three cylindrical wells of approximately 2 mm in diameter and depth were created on the surface of the base using an indenter. The wells were positioned on the outer edge of the phantom and separated by a gap of ≈10 mm to avoid any overlap of signals. Cell pellets of the treated and untreated cells were homogenously dispersed in 20 *μ*L low gelling temperature agarose (Sigma, 1% w/v in PBS) at 37 °C and placed in the cylindrical wells in the bottom layer of solidified agar. On solidification of the pellets, 15 ml of agar solution, with the same concentration of agar and intralipid® as the bottom layer was used to cover the inclusions. Cells used in the MRI experiments were grown in standard DMEM medium or medium containing iron in the form of the superparamagnetic iron oxide contrast agent Endorem (Guerbet, France) at 0.5 *μ*g/ml during the 72 h incubation with DMSO or drugs to increase iron chelation. At the end of each experiment cells were harvested by trypsinization and washed twice in PBS and a dry pellet of ≈6 million cells transferred to a 0.75 ml eppendorf tube for MRI imaging.

### PAI data acquisition and processing

Cell pellet-containing phantoms were imaged using the MSOT inVision 256TF system (iThera Medical, Germany). The phantoms were horizontally positioned in the thin polyethylene membrane holder for MSOT imaging ensuring cell pellets positioning within the 270° angle curvature of the MSOT transducer for homogenous illumination. The holder was submerged in distilled water at 34 °C and the phantom was positioned in the centre of the MSOT transducer (partial ring array of 256 elements, each with a centre frequency of 5 MHz and a bandwidth of greater than 60%) and imaged at wavelengths over a range of 670 nm to 950 nm, at intervals of 10 nm (10 pulses per wavelength, 10 nanosecond laser pulses, pulse repetition interval of 100 ms). With a maximum energy of the laser being approximately 70 mJ, the resulting surface fluence on the phantom was calculated to be approximately 14 mJ/cm^2^. The experimental and instrument settings were held constant so that the signal strengths of various inclusions, across independent measurements could be compared. The MSOT images were reconstructed using the interpolated model matrix inversion algorithm^53^. The mean and standard deviation of the SBR were calculated from regions of interests enclosing the entire cell-inclusions and background, across the wavelength range, using the MSOT inVision software 3.8 (iThera Medical, Germany). Statistical significance of any differences in the peak SBR at 670 nm and the area under the curve of the SBR plot, between the treated and control cells, was determined using Student’s t-test.

### MRI data acquisition and processing

Relaxometry studies were performed on a 7T horizontal bore MRI system (Bruker Instruments, Germany) using a 40 mm circularly polarised, transmit receive volume coil and the cell pellet-containing tubes immersed in a 50 ml water-filled test tube. Data processing was carried out in Paravision 6.0 (Bruker, Germany). A first set of *T*_2_ weighted images were acquired using a rapid acquisition with refocused echoes (RARE) sequence, with *TE*_*e*_ *ff* = 36 ms, TR = 4.5 s, RARE factor = 8, 30 contiguous 1-mm thick transverse slices, 1 average, matrix size 128 × 128 over a 3 × 3 cm^2^ field of view. These images were used for planning the subsequent measurement, which included optimization of the local field homogeneity using Mapshim algorithm and the measurement of:

- T_2_* maps were calculated from a multi-gradient echo (MGE) sequence (8 readout echoes: TR = 200 ms, first TE/ΔTE = 3 ms/3 ms, minimum echo spacing 1.82 ms, flip angle 60°, 5 slices, slice thickness 1 mm, 8 averages, 128 × 128 scan matrix, 3 × 3 cm^2^ field of view).
- *T*_1_/*T*_2_ relaxation curves were calculated from a rapid inversion recovery (IR) True Fast Imaging with Steady-state Precession (TrueFISP) sequence (TE = 1.9 ms, TR = 3.8 ms, 50 inversion times spaced 61.16 ms apart, initial inversion time 25 ms, total scan repetition time of 10 s, flip angle 60°, one 0.5 mm slice, 8 averages of a matrix size of 128 × 128 over a 3 × 3 cm^2^ field of view).
- *T*_1_, *T*_2_ and ${T}_{2}^{\ast }$ maps were calculated from regions of interest using the in-built post-acquisition Image Sequence Analysis (ISA) tool. The mean and standard deviation of the ratio of the measured relaxation times of the treated cells to that in the controls was calculated and plotted using Prism™ version 7 (GraphPad, San Diego).

For display purposes *T*_1_ colour coded maps were generated using ImageView Software v5.1.2 (IDL, ITT, US).

The CV of the ratio of imaging signal in the treated cells to that in the controls, was estimated, to acquire an indication of the relative sensitivity of the two techniques. The CV was calculated as the ratio of the standard deviation to the mean.

### Western blotting

Changes in protein expression of the melanoma cell differentiation markers tyrosinase and gp100 were assessed with western blotting using standard conditions. The primary antibodies used were rabbit anti-tyrosinase (Abcam; Cambridge, UK), anti-gp100 (Abcam; Cambridge, UK), anti-P-ERK1/2, anti-ERK1/2 (Cell signaling Technology; Danvers, MA, USA), and mouse anti-Hsp70 (Stressgen Bioreargents; Michigan, USA) and anti-*β* actin (Chemicon; Hampshire, UK). The secondary antibodies used were anti-mouse (Dako A/S, Glostrup, Denmark) and anti-rabbit (GE Healthcare Life Sciences; Buckinghamshire, UK). Antibody-target protein interactions were visualised by enhanced chemiluminescence (GE Healthcare Life Sciences).

### Melanin quantification

Melanin content was measured following a previously published protocol^54^. Briefly, after 72 h treatment with 50 nM 17-AAG or 1 *μ*M vemurafenib, 1 million cells were solubilised in 100 *μ*l of 1 M NaOH and samples incubated at 60 °C for 1 h and vortexed to solubilise the melanin. Absorbance at 405 nm was measured and normalised to protein concentration estimated in the same sample using the Bradford method.

### Statistical Analysis

The statistical significance of the results was assessed using Student’s unpaired 2-tailed t-test with p values ≤ 0.05 considered to be significant. Data are represented as the mean ± standard error of the mean.
